# Inflorescence stem grafting made easy in Arabidopsis

**DOI:** 10.1186/1746-4811-8-50

**Published:** 2012-12-19

**Authors:** Nazia Nisar, Shelley Verma, Barry J Pogson, Christopher I Cazzonelli

**Affiliations:** 1 Australian Research Council Centre of Excellence in Plant Energy Biology, College of Medicine, Biology and Environment, Research School of Biology, The Australian National University, Canberra, ACT 0200, Australia

**Keywords:** Grafting, Wedge, Plant development, Inflorescence stem, Systemic, Signal, Molecule, Transmission, Arabidopsis, Scion

## Abstract

**Background:**

Plant grafting techniques have deepened our understanding of the signals facilitating communication between the root and shoot, as well as between shoot and reproductive organs. Transmissible signalling molecules can include hormones, peptides, proteins and metabolites: some of which travel long distances to communicate stress, nutrient status, disease and developmental events. While hypocotyl micrografting techniques have been successfully established for Arabidopsis to explore root to shoot communications, inflorescence grafting in Arabidopsis has not been exploited to the same extent. Two different strategies (horizontal and wedge-style inflorescence grafting) have been developed to explore long distance signalling between the shoot and reproductive organs. We developed a robust wedge-cleft grafting method, with success rates greater than 87%, by developing better tissue contact between the stems from the inflorescence scion and rootstock. We describe how to perform a successful inflorescence stem graft that allows for reproducible translocation experiments into the physiological, developmental and molecular aspects of long distance signalling events that promote reproduction.

**Results:**

Wedge grafts of the Arabidopsis inflorescence stem were supported with silicone tubing and further sealed with parafilm to maintain the vascular flow of nutrients to the shoot and reproductive tissues. Nearly all (87%) grafted plants formed a strong union between the scion and rootstock. The success of grafting was scored using an inflorescence growth assay based upon the growth of primary stem. Repeated pruning produced new cauline tissues, healthy flowers and reproductive siliques, which indicates a healthy flow of nutrients from the rootstock. Removal of the silicone tubing showed a tightly fused wedge graft junction with callus proliferation. Histological staining of sections through the graft junction demonstrated the differentiation of newly formed vascular connections, parenchyma tissue and lignin accumulation, supporting the presumed success of the graft union between two sections of the primary inflorescence stem.

**Conclusions:**

We describe a simple and reliable method for grafting sections of an Arabidopsis inflorescence stem. This step-by-step protocol facilitates laboratories without grafting experience to further explore the molecular and chemical signalling which coordinates communications between the shoot and reproductive tissues.

## Background

Grafting is a traditional method for uniting one part (scion) of a plant (e.g. shoot or bud) with the root system of another. It has become a fundamental method for studying physiological, genetic and molecular aspects of long distance signalling events. There are many grafting techniques described, of which some are widely used in agriculture as a mean to facilitate asexual propagation. Advantages are to induce dwarfing and fruitfulness, avoid juvenility, promote sturdiness [1,2] as well as facilitate domestication of both woody and non woody plant species [3,4]. In the laboratory, grafting for most dicot species is now a well-established practice widely used to study plant nutrition [5], disease resistance against phloem associated pathogens [6,7], apical dominance and shoot branching [8,9].

Grafting has provided a powerful approach to combine diverse genotypes and investigate the transport of various long-distance signalling molecules [8]. The technique of grafting has demonstrated that 1) the transfer of tuberigen stimulates tuberization in potato [10-12], 2) autoregulation of nodulation is regulated via long-distance shoot and root derived signals, [13-16], 3) allocation of nutrients by the Arabidopsis Na^+^ transporter AtHKT1 functions in the root to control Na^+^ accumulation in the shoot [17-20] and that the phloem mobile miRNA that acts as long-distance signal regulating phosphate homeostasis in Arabidopsis [18,19], 4) transport of phytohormone growth molecules such as the movement of cytokinin from roots to shoots [21] and translocation of strigolactone from root to shoot which inhibits shoot branching [22,23], 5) the movement of secondary metabolites such as alkaloids [24] and 6) mobile flowering signals synthesized in the leaves can stimulate flowering within the shoot apical meristem [25-28].

Hypocotyl micrografting techniques have been successfully used to study developmental and physiological processes between the root and shoot [2,8,29-31], while the inflorescence meristem grafting methods have generally been used to investigate the nature of signals coordinating floral reproductive growth [32-36]. Indeed small RNAs have been shown to move across the junctions from hypocotyl and inflorescence grafts, most likely via the plasmodesmata rather than the phloem [37-39]. Experiments investigating the movement of mobile signals that control apical dominance (cytokinins and auxin), nutrient uptake (phosphorus) and cellular differentiation (small RNAs) are key research areas that can benefit from the inflorescence grafting technique. Given that this technique investigates a post-floral transition, it provides a unique system to discover signals mediating reproductive development.”

This paper describes a protocol for efficient wedge-style grafting of the primary inflorescence scion to the rootstock. We define the location, timing and preparation of the floral stems for grafting. An inflorescence growth assay was developed to determine the healthiest of grafts. Histological studies confirmed the differentiation of vascular connections as well as a successful graft union.

## Results and discussion

### A robust and highly efficient grafting method for arabidopsis

Preliminary homografting experiments were carried out using young primary inflorescence stems of uniform age, length, and diameter using the model plant, *Arabidopsis thaliana*. Both horizontal [35] and wedge-style [34] grafting techniques proved less successful then previously reported. Difficulties were encountered in maintaining hydraulic turgor as well as providing structural support to the grafted stem sections. For the horizontal grafts we found that by sliding silicone tubing over the graft junction and sealing with either parafilm or Petroleum Jelly (Vaseline) provided structural support as well as minimise scion transpiration allowing reproductive tissues to survive until the graft union re-establishes xylem and phloem continuity. However, within one week of growth the grafted scions began to wilt showing signs of necrosis, most likely due to poor contact between the grafted stem sections. It is essential that sufficient contact between the stem of the scion and root stock is maintained in order to promote the development of vascular connections, which allow the flow of nutrients through the stem to the reproductive organs.

**Figure 1:**
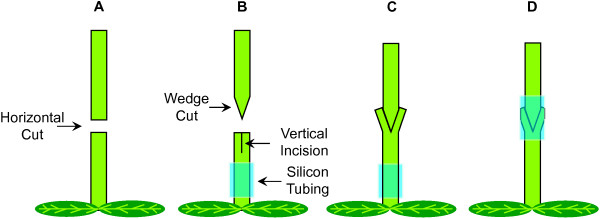
**Schematic illustration displaying an Arabidopsis wedge grafting technique.** (**A**) Horizontal cut of the floral stem. (**B**) The floral scion was trimmed to form a wedge and the floral stem stock vertically cut to form a donor wedge in which to insert the floral scion (**C**), before sliding a tightly fitted silicone tube (blue) over the wedge junction (**D**).

**Figure 2:**
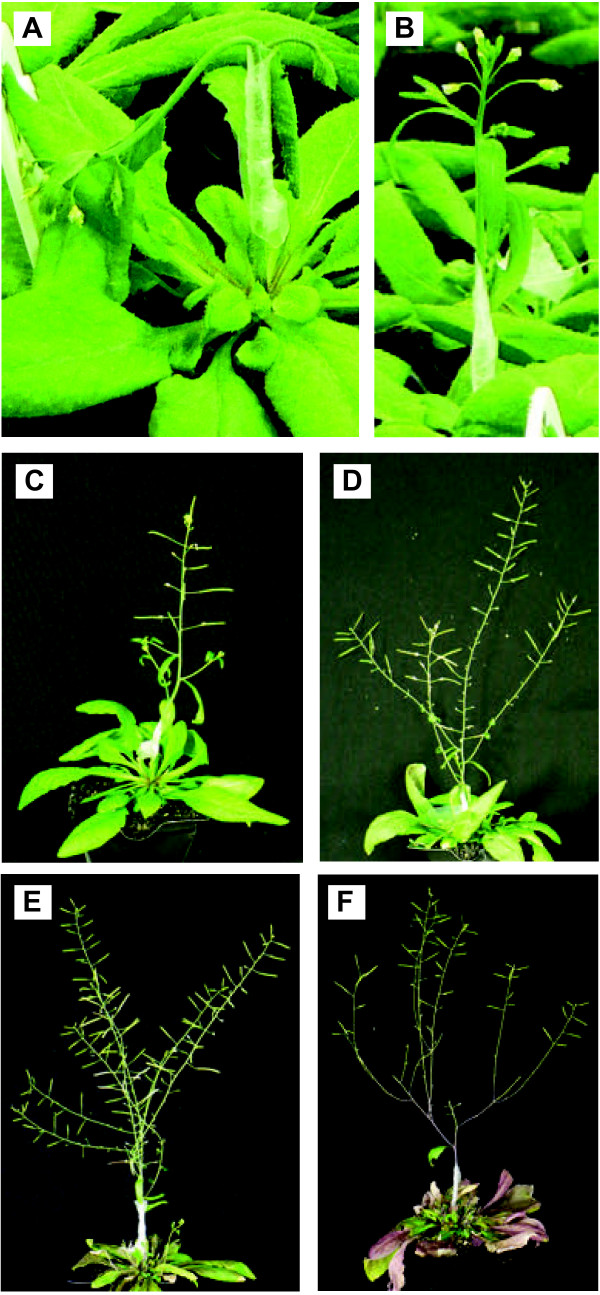
**Growth habits of grafted inflorescence stems from *A*. *thaliana*.** Floral stem grafts wilt immediately after sealing the graft (**A**) and if successful recover within 24 hours by standing upright (**B**). Successful floral stem grafts will maintain shoot apical dominance (**C**; 7 DAGr) and develop extended cauline shoot branches (**D**; 14 DAGr) with healthy reproductive siliques (**E**; 22 DAGr). A grafted plant was pruned to remove all reproductive siliques and at the cauline nodes, new cauline leaves and shoots were produced leading to the formation of new reproductive siliques (**F**; 60 DAGr).

Next we performed the wedge-style graft, reported to have a much higher success rate, potentially due to greater contact area between tissues at the graft junction, but difficulties were encountered when attempting to stabilise the grafted stem sections with thin glass splints [34]. Instead, a 1.5 cm length of silicone tubing placed over the wedge provided excellent structural support to the primary inflorescence and ensured good contact at the junction of the wedge. The silicone tubing was flexible and did not appear to restrict inflorescence stem growth. The silicone supported graft junction was further sealed with parafilm to prevent desiccation and maintain turgor (Figure 1). Immediately following grafting the grafted scions began to wilt (Figure 2A) and within 24 hrs the grafted scions regain turgor and stand upright (Figure 2B, 1DAGr). Without any prior experience, a 35% success rate was achieved using this technique in a pilot experiment. Typically, greater than 87% (n = 103) success rates were achieved by several experienced researchers in our group, highlighting a very simple, efficient and robust wedge-style grafting technique (Figure 1).

### A non-destructive inflorescence growth assay for identifying healthy grafts

The success of grafting was determined by monitoring scion growth and development: processes that require the transport of water, nutrients and signalling molecules. Previously, grafting had been considered successful when scions remained alive on top of the rootstock [34]. Here we further redefine that a successful floral stem graft should 1) be able to maintain shoot apical dominance as evidenced by a taller primary stem (Figure 2C; 7 DAGr), 2) grow healthy cauline shoot branches which continue to develop (Figure 2D; 14 DAGr) and 3) produce fertile reproductive siliques (Figure 2E; 22 DAGr). The most definitive measure of success of a grafted plant is by pruning (i.e. remove cauline shoot branches and reproductive siliques), which promotes the growth of new cauline leaf and shoot tissues eventually leading to the formation of new reproductive siliques (Figure 2F; 60 DAGr).

**Figure 3:**
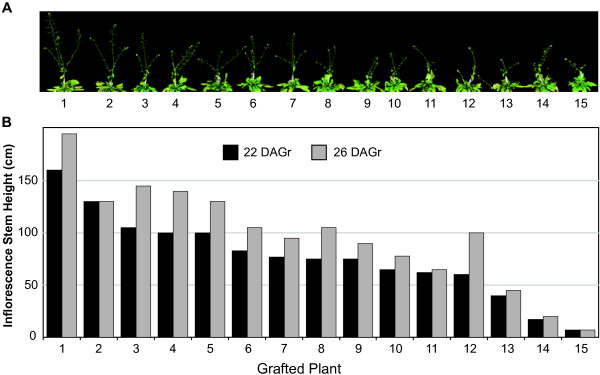
**Evaluation of the growth status of successful grafts.** (**A**) Images of 15 healthy grafted plants showing a range in inflorescence growth habits. (**B**) Inflorescence stem height (cm) of 15 grafted plants measured 22 and 26 DAGr. The stem height reflects the distance between the first cauline node and top of the primary inflorescence flower. The numbers below the images (**A**) and on the x-axis of the graph (**B**) identify the grafted plant being tested.

We identified some “successful” grafts that remained viable, but, the scion stem failed to grow and maintain apical dominance, as evident by longer cauline branches relative to the primary rosette stem (Figure 3A; plants 13–15), which is indicative of an incomplete union. The number of cauline branches (~2-3) was similar for the “successful” grafts (Figure 3A). Therefore, we devised a non-destructive growth method for revealing fully successful and healthy grafted plants by quantifying the distance between the oldest (lowest on the scion) cauline node and top of the primary inflorescence scion stem. We measured the inflorescence stem height from 15 healthy and “successful” looking homografted plants (22 and 26 DAGr) and observed 12 grafted plants that showed sufficient growth of the scion stem, and also maintained control over apical dominance, as evidenced by shorter cauline shoot branches compared to the primary stem (Figure 3B). However, only 10 of the apically dominant grafted plants continued shoot growth between 22 and 26 days after grafting (Figure 3B), revealing that this non-destructive growth assay is a viable means by which to select the best grafted plants suitable for physiological and molecular investigations.

### Anatomy of graft union formation at the wedge junction

**Figure 4:**
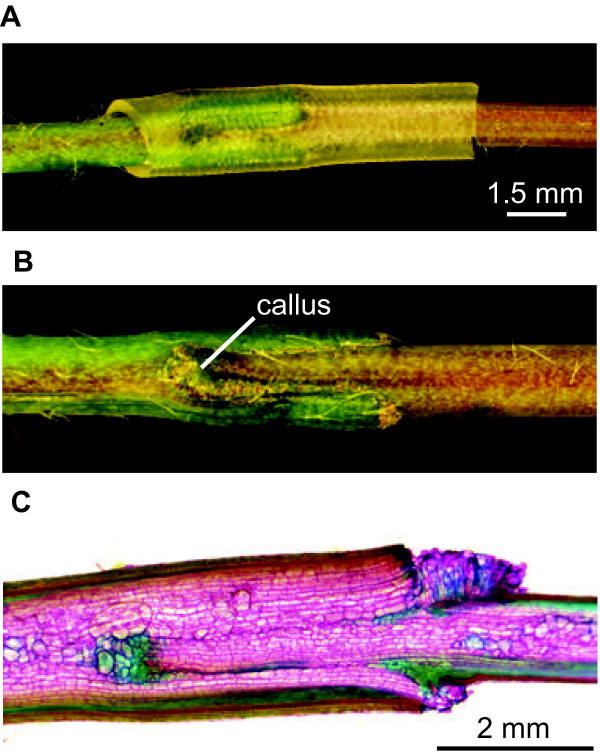
**Longitudinal views of a grafted inflorescence *A. thaliana *stem.** A floral stem graft showing expansion of the silicone tubing surrounding the wedge junction (**A**) and grafted stem tissues fused tightly in the absence of the silicone tubing (**B**). (**C** and **D**) A longitudinal section through the floral stem graft shows lignified tissues (blue-green) and non-lignified (purple-red) tissues surrounding the grafted wedge.

**Figure 5:**
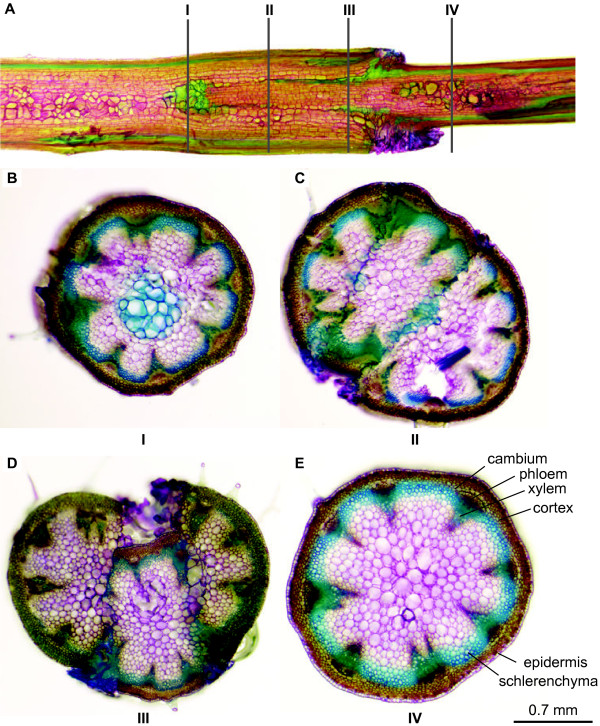
**Cross-section of an inflorescence stem of *A. thaliana *stained with toluidine blue.** (**A**) Side view of the wedge graft junction showing the positions of 4 transverse sections. (**B**-**E**) Transverse sections taken from the bottom (**B**), middle (**C**) and top (**D**) of the wedge-graft as well as from an ungrafted floral stem section taken above the wedge-graft (**E**).

We analysed the integrity of graft union formation to confirm that vascular connections were established between the scion and stem from the root stock. The formation of regenerative xylem vessels following wounding occurs mostly in young inflorescence stems (e.g. 2 to 4 day-old) [34] and indeed the lower section (2–4 cm above the rosette) of a young primary inflorescence stem was found to best suited for wedge grafting. Removal of the parafilm covering the silicone tube revealed a sealed graft junction (Figure 4A) that remained tightly connected even after the silicone tube was removed (Figure 4B). Callus proliferation was clearly observed surrounding the wedge junction providing a good indication of regenerative growth (Figure 4B). Histologic analysis of a longitudinal section through the wedge graft showed the differentiation of new vascular tissues within the scion, which were associated with lignin accumulation (blue-green cells) and as well as a tight vascular graft union formation between the scion and the stock (Figure 4C). Transverse sections through the wedge graft revealed a small deposit of lignin at the tip of the scion graft (Figure 5A and 5B), formation of a necrotic plate (Figure 5C, 5D), differentiation of parenchymal cells and vascular tissues (Figure 5D) when compared to a section of wild type stem (Figure 5E). The histological data supports the formation of a successful graft union.

In summary, we have developed a robust grafting method for Arabidopsis that is efficient and easily scaled for high throughput studies. A series of tips ensure a high success rate (Table 1) and the development of the non-destructive inflorescence growth assay provides a simple measure by which to determine the healthiest and most successful grafted plants amenable to physiological, metabolic and molecular studies interested in dissecting the signalling pathways controlling reproductive growth in Arabidopsis.

**Table 1:** Technical tricks for successful wedge-style inflorescence grafting in Arabidopsis

**Step**	**Description of method**	**Tips and tricks**
**1**	Plant Growth Before Grafting	Vernalise seedlings to synchronise the initiation of floral meristem development.
		Use young inflorescence stems of uniform age, length (<10 cm), and diameter (1.5 to 3 mm).
**2**	Horizontal Excision of Stem	Use flexible razor blade to cut the floral stem below the cauline node (2 cm above the shoot meristem) and immediately place scion in sterile water.
**3**	Preparation of Root Stock Stem	Slide 1 cm of soft and flexible silicone tubing (sizes range from 1–3 mm ID) over the root stock stem.
		Cut vertical incision into the root stock stem.
**4**	Preparation of Scion Stem	Trim the scion stem using flexible razor blade into a wedge.
**5**	Join and Support Grafted Stems	Place scion wedge into root stock stem incision.
		Slide the silicone tubing over the graft junction.
		Inject sterile water on top of silicone tube to maintain turgor.
		Pre-stretch parafilm and wrap over the graft junction supported by silicone tube so as to avoid desiccation.
**6**	Plant Growth After Grafting	Maintain high humidity (70%) for 1–2 weeks.
		Slowly acclimate grafted plants to normal growth conditions.
		Clip new rosette shoot growth to promote further development of the primary grafted floral stem.
		Remove meristems showing reproductive siliques to promote new cauline shoot growth of new tissues.
**7**	Evaluation of Grafting Success	Grafting was considered successful when scions remained green and health on top of the rootstock as evident by the maintenance of apical dominance, development of new cauline leaves and branches, as well as production of reproductive siliques.
		Use the inflorescence growth assay to select the best and most successful grafted plants for further analysis.

## Materials

### Planting and growth conditions

Dried seeds were sown onto DEBCO seed raising mixture, fertilized with 3 g/L of mini Osmocote and cold-stratified at 4°C in the dark for three days to synchronise germination. Seeds were germinated in the light (16 hrs, 21°C, 55% humidity, 150 μmol m^-2^ s^-1^ irradiance), irrigated twice a week with water and grown under these conditions unless otherwise stated. In order to synchronise inflorescence meristem initiation, germinated seedlings (7 DAG) were vernalised for four weeks at 4°C before returning seedlings to the growth conditions described above. Vernalization may not need to be performed if all plant genotypes flower simultaneously. Reducing the day length will slow growth and produce larger rosettes with thicker inflorescence meristems, thereby facilitating preparation of the scion and root stock for wedge style grafting.

### Histology and microscopy

Plant samples were stained with aqueous 0.01% Toluidine Blue-O for 1 minute and rinsed in water. Fresh tissue specimens were placed on glass slides, covered with a glass coverslip and water used to fill the airspaces and maintain cell turgor. Slides were examined under a Leica MZ16FA Fluorescence Stereomicroscope and representative sections photographed.

### Reagents and consumables

· Double edge safety razor blades (fine stainless steel, platinum coated to enhance sharpness)

· 1 cc/mL Plastic syringe

· Silicone Tubing 1.5 mm ID × 2.3 mm OD

· Silicone Tubing 2.0 mm ID × 2.8 mm OD

· Silicone Tubing 2.5 mm ID × 3.3 mm OD

· Silicone Tubing 3.0 mm ID × 3.8 mm OD

· Petri dishes

· Clear plastic humidity chamber

· Plastic paraffin film (Parafilm)

· Sterile water

## Protocol

### Arabidopsis wedge style grafting

Grafting was performed when the primary inflorescence meristem reached a height of 5 to 10 cm above the rosette and floral buds became clearly visible). It is important to grow a large number of plants (>30) in order to ensure that floral stems of a similar thickness can be matched.

1) Remove the floral bolt from the rootstock (leave 3-5 cm of the inflorescence stem on the root stock) by making a horizontal cut (with a two edge razor blade) and immediately place the scion in a petri dish containing water to prevent air entering the vascular stream. If performing many grafts simultaneously, place a small droplet of water on the rootstock stem to prevent any desiccation.

2) Place a 1.5 cm long section of silicone tubing (1–4 mm internal diameter depending upon thickness of floral bolt) over the shoot stump on the root stock. The tubing should have a slightly larger internal diameter than the stem to be grafted allowing the tubing to slip up over the graft junction.

3) Make a 1 cm long median incision along the length of the shoot stump on the root stock and immediately add a drop of water inside the incision using a syringe, creating a v-shape wedge. This will keep the incision moist and promote an efficient union between the scion and rootstock.

4) Cut the horizontally-severed floral stem into a long v-shape wedge under water and position inside the incision of the root stock stem. The wedge graft will provide greater contact between vascular as well as improve surface tension.

5) Slide the silicone tubing over the wedge graft to secure contact between the scion and root stock. The silicone tubing will provide vertical support to the upper floral scion. Use a syringe to inject water above the silicone tubing and onto the floral stem stump in order to prevent desiccation.

6) The graft junction was further sealed by wrapping with parafilm (1 cm above and below the silicone tubing) to maintain hydraulic turgor and prevent desiccation. The parafilm needs to be stretched before application to generate a slightly adhesive surface making it easier to seal around the graft junction.

7) Cover the grafted plants with a clear plastic humidity chamber in order to maintain a humid environment. If you have a growth chamber, adjust to 70% humidity.

8) Water the grafted plants regularly to help maintain a humid environment and begin to acclimate to normal growing conditions within two weeks of performing the grafts.

## Abbreviations

DAG: Days after germination; DAGr: Days after grafting; ID: Internal diameter; OD: Outside diameter.

## Competing interests

The authors declare that they have no competing interests.

## Authors’ contributions

CIC and BJP planned the research. NN, SV and CIC developed the grafting techniques and contributed to writing the manuscript as well as preparing figures. All authors read and approved the final manuscript.
